# Autoimmune Antibodies in Orthostatic Intolerance Syndromes

**DOI:** 10.33549/physiolres.935504

**Published:** 2025-04-01

**Authors:** Monika LUKÁČOVÁ, Peter MITRO, Zora LAZÚROVÁ, Emília HIJOVÁ, Izabela BERTKOVÁ, Kristína VALOVÁ

**Affiliations:** 1 1^st^ Department of Cardiology, VUSCH, P. J. Safarik University, Kosice, Slovakia; 2 Centre for Clinical and Preclinical Research Medipark, P. J. Safarik University, Kosice, Slovakia

**Keywords:** Postural tachycardia syndrome, Vasovagal syncope, Orthostatic intolerance, Autoimmunity, Autoantibody

## Abstract

Orthostatic intolerance (OI) is defined as the development of characteristic symptoms while standing, which significantly improve by recumbency. The most common forms are vasovagal syncope (VVS), orthostatic hypotension, and postural orthostatic tachycardia syndrome (POTS). Lately, there has been a growing body of evidence that autoimmunity may play a role in the pathophysiology of orthostatic intolerance syndromes. The aim was to compare the presence and levels of autoimmune autoantibodies in patients with POTS, VVS syncope, and the control group. Altogether, 61 patients with symptoms of orthostatic intolerance were evaluated in this study - 19 POTS patients and 42 VVS patients. The control group contained 22 patients with no signs of orthostatic intolerance. We evaluated levels of autoantibodies against three subtypes of G-protein coupled adrenergic receptor (alpha-1 and beta-1,2 adrenergic receptors), type 4 of muscarinic acetylcholine receptor, and angiotensin II type 1 receptor. We compared the levels between the three patient groups. Significantly higher levels of angiotensin II type 1 receptor (AT1R) autoantibodies were found in the POTS group compared with controls (0.67± 0.35 ng/ml vs. 0.38±0.32 ng/ml, p=0.008). There was no significant difference in AT1R antibodies between the VVS and control groups (0.46±0.34 ng/ml vs 0.38±0.32 ng/ml, p= 0.38). Autoantibody concentration against ADRA1, ADRB1, ADRB2, and M4R were not significantly different between the groups. Autoimmune mechanisms may lead to abnormal regulation of the renin-angiotensin-aldosterone system and may contribute to the pathophysiology of POTS.

## Introduction

Orthostatic intolerance is defined as developing characteristic symptoms while standing, which significantly improve by recumbency. These symptoms include light-headedness, fatigue, palpitation, syncope, chest discomfort, headache, nausea, dizziness, abdominal pain, sweating, neurocognitive impairment, anxiety [1]. The most common forms of orthostatic intolerance are vasovagal syncope (VVS), orthostatic hypotension (OH), and postural orthostatic tachycardia syndrome (POTS) [2].

Vasovagal syncope is the most common form of orthostatic intolerance syndromes, which describes a syncopal episode (transient loss of consciousness) caused by intermittent inappropriate control of cardiovascular reflexes resulting in hypotension and/or bradycardia [3].

Orthostatic hypotension is a condition characterized by a drop in systolic blood pressure ≥20 mmHg or a drop in diastolic blood pressure ≥10 mmHg after an orthostatic challenge [4].

Postural orthostatic tachycardia syndrome is characterized by a sustained increase in heart rate ≥30 bpm (≥ 40 bpm for adolescents aged 12–19 years) during 10 minutes of standing in the absence of orthostatic hypotension with associated symptoms of orthostatic intolerance. Symptom duration should be at least three months and there should be no other condition that would explain sinus tachycardia [4].POTS etiology is heterogeneous. Multiple mechanisms have been considered responsible for the development of POTS - peripheral denervation, hyperadrenergic state, hypovolemia, viral illness, mast cell activation, and, recently immune dysregulation [5,6].

Lately, there has been a lot of interest in the role of autoimmunity in orthostatic intolerance syndromes, especially in POTS. The direct link has not been established, yet autoimmune diseases and POTS share some clinical features - female predominance, post-viral onset, or the presence of various autoantibodies [4,6]. To date, there have been some studies concerning autoimmune antibodies against adrenergic, cholinergic, and angiotensin II type I receptors in POTS patients with different results, and very few available studies regarding these autoimmune antibodies in VVS patients [7,8].

In 2012, Wang *et al*. were the first to imply autoimmunity as a possible etiology of POTS by detecting autoimmune IgG antibodies in patients with POTS [9]. Later, in 2014, Li *et al*. documented specific antibodies against receptors in the autonomic nervous system in POTS patients, specifically autoantibodies against adrenergic alpha-1 receptors (ADRA1) and autoantibodies against beta-1 (ADRB1) and beta-2 adrenergic receptors (ADRB2) [10]. Studies suggest that antagonistic antibodies to alpha-1 adrenergic receptors could result in the failure of peripheral vasoconstriction during orthostatic demand. Agonistic beta-1 and two adrenergic antibodies may lead to compensatory tachycardia in POTS patients [11]. Alpha-2 receptor agonists seem to have an impact on body fluid control through aquaporin-2 (channel binding vasopressin) - these agonists potentially increase free water clearance secondary to inhibition of vasopressin [12]. Muscarinic cholinergic receptor antibodies possibly act as vasodilators. There are five different epitopes - M1–M5 [13].

Angiotensin II type 1 receptor antibodies have also been studied in patients with POTS and may act by disrupting systemic vasoconstriction in response to upright position [4,12].

Supposed autoimmune mechanisms in the pathophysiology of other orthostatic intolerance syndromes are lacking robust evidence. Li *et al*. documented that 5 out of 6 subjects with idiopathic orthostatic hypotension had ADRB2 autoantibodies [14]. Lu *et al*. found ADRB1, ADRB2, M2R, and M3R autoantibodies in idiopathic orthostatic hypotension patients [12].

The aim of this study was to assess the possible role of various autoimmune autoantibodies - against G-protein coupled receptors - adrenergic, cholinergic receptors, and angiotensin II type 1 receptor - in the pathogenesis of POTS and VVS. The aim was to evaluate the levels of autoantibodies and compare the levels between 3 patient groups - the patients with POTS, VVS, and the control group.

## Methods

Altogether, 61 patients with symptoms of orthostatic intolerance (19 POTS patients, 42 VVS patients) and 22 healthy subjects with no symptoms of orthostatic intolerance and/or autoimmune disease were evaluated in this study.

Inclusion criteria were symptoms of orthostatic intolerance with a diagnosis of POTS or vasovagal syncope confirmed by head-up tilt test.

Exclusion criteria were abnormal electrocardiogram, structural heart disease, neurodegenerative diseases, peripheral neuropathy, 1-type diabetes mellitus, poorly compensated thyroid gland diseases, use of cardioactive drugs or severe anemia.

The most common comorbidities were arterial hypertension and thyroid gland diseases. Patient group demographics and comorbidities are shown in Table 1.

All patients underwent head-up tilt test (HUTT), which was performed according to Italian protocol, in 60 60-degree position (20 minutes of passive phase, 15 minutes of active phase provoked by sublingual 300 ug nitroglycerin). All cardioactive medications were withheld for 3 days.

The diagnosis of POTS was made in the presence of a sustained increase in heart rate ≥ 30 bpm (at least two measurements 1 minute apart) during 10 minutes of standing in the absence of orthostatic hypotension. BP was measured by brachial oscillometric blood pressure cuff; heart rate was measured by continuous ECG monitoring. The healthy individuals did not undergo tilt testing since there was no indication. The diagnosis of VVS was made according to the VASIS classification (The Vasovagal Syncope International Study), the most accepted one to define the type of response to tilt testing [15].

Blood samples were collected from the antecubital vein before HUTT. Samples were left to clot for two hours at room temperature before centrifugation for 15 minutes at 1000 xg, and then serum was removed and stored at −80 °C until analysis.

The levels of autoantibodies against three subtypes of G-protein coupled adrenergic receptor (alpha-1 and beta-1,2 adrenergic receptors), one subtype of G-protein coupled muscarinic acetylcholine receptor (type 4 muscarinic receptor), and angiotensin II type 1 receptor were evaluated.

All endpoints were measured by ELISA methods, except M4R which was measured by EIA methods as follows: Human angiotensin II type 1 receptor (AT1R, Cat. No. MBS267431 by My BioSource.com), Human anti alpha-1 adrenergic receptor (ADRA 1 IgG, Cat. No. MBS7207754 by My BioSource.com), Human beta-1 adrenergic receptor antibody IgG (ADRB1-Ab IgG, Cat. No. MBS7269092 by My BioSource.Com), Human beta-2 adrenergic receptor antibody IgG (ADRB2-Ab IgG, Cat. No. MBS7269093 by My BioSource.com), Anti muscarinic cholinergic receptor four antibodies (M4 by Eagle Bioscience, Inc.). The final values of each parameter were measured on the Synergy H4 multi-plate reader (BioTek Instruments, Inc. USA).

For statistical analysis, the MedCalc Statistical Software version 19.2.6 (MedCalc Software bv, Ostend, Belgium) was used. The antibody levels were expressed as arithmetical mean± standard deviation.

To test the differences in parametric continuous variables, ANOVA test was performed, for post-hoc analysis, the Tukey-Kramer test was used (3 multiple comparisons - POTS vs. Controls, POTS vs. VVS, VVS vs. Controls). For abnormal distribution and equal variances, the non-parametric Kruskal-Wallis test with Dunn comparison was used, in case of variables with unequal variances, the Welch’s ANOVA test was performed. For categorical variables, we used Pearson’s chi-squared test. The results were considered statistically significant if the p-value was ≤0.05.

## Results

Autoantibody concentration against ADRA1, ADRB1, ADRB2, and M4R were not significantly different between groups. The only difference was observed in angiotensin II type 1 receptor (AT1R) autoantibodies (p=0.015).

Significantly higher levels of angiotensin II type 1 receptor (AT1R) autoantibodies were in the POTS group compared with controls (0.67± 0.35 vs. 0.38±0.32, p=0.008). There was no significant difference in AT1R antibodies between the VVS group and the control group (0.46±0.34, vs 0.38±0.32 p=0.38). The levels of autoantibodies and p-values are shown in Table 2.

**Fig. 1:**
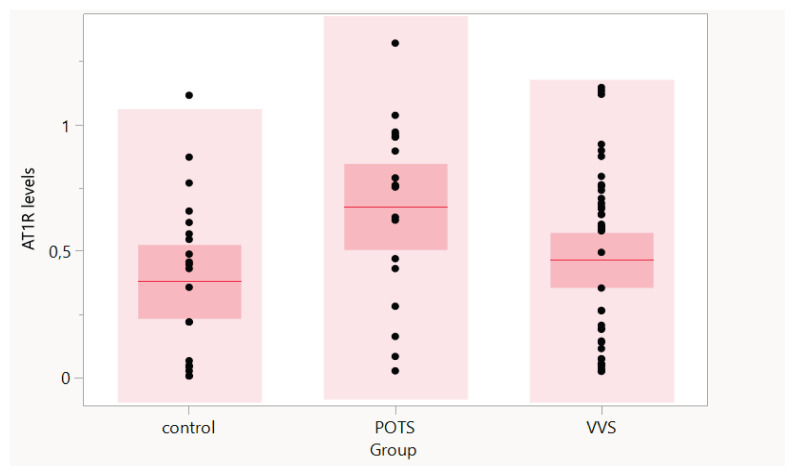
AT1R antibodies levels in different study groups. AT1R - angiotensin II type 1 receptor; POTS - postural orthostatic intolerance syndrome; VVS - vasovagal syncope. The red line represents the group average, the darker pink interval represents the confidence interval. (Graph was obtained from MedCalc Statistical Software)

A graphical display of levels of autoantibodies against AT1R according to the different groups is in Figure 1.

There was a trend to higher levels of AT1R antibodies in men with POTS compared to women with POTS, but the difference was not statistically significant. There was also a trend to higher levels of M4R antibodies in women with POTS compared to men with POTS, but without statistical significance (Table 3).

There were no significant differences in the levels of antibodies between women and men in patients with VVS (Table 4).

## Discussion

The aim of this study was to compare the concentrations of adrenergic, cholinergic and angiotensin receptor autoantibodies against autonomic nervous system receptors between the POTS group, VVS group and the control group. Our hypothesis was that POTS patients would have higher levels of autoantibodies compared with controls and VVS group.

The underlying pathophysiology of POTS is not fully understood. A growing body of evidence suggests that POTS may be an autoimmune disorder. POTS patients have at least 1 elevated G-protein coupled adrenergic antibody [13]. Another orthostatic intolerance syndrome VVS is not considered to be an autoimmune disease.

In our study, we found significantly higher levels of autoantibodies against angiotensin II type 1 receptor in POTS patients compared with the VVS group and the control group. This result is consistent with previous research. In a much smaller study Yu et al demonstrated significant AT1R activity in 12 out of 17 POTS subjects and no significant AT1R activity in the subjects with vasovagal syncope (6 patients) and the control group (10 patients) [7].

In the renin-angiotensin-aldosterone humoral system, renin converts angiotensinogen to angiotensin I, angiotensin-converting enzyme (ACE) than converts angiotensin I (AT I) to angiotensin II (AT II) which stimulates aldosterone production via type 1 angiotensin receptor (AT1R). Angiotensin II is degraded into angiotensin I through angiotensin-converting enzyme 2 (ACE2). Angiotensin II is a potent vasoconstrictor and important regulator of the circulating plasma volume [16]. According to Mustafa et al, POTS patients have increased levels of AT II and reduced activity of ACE2 (enzyme degrading angiotensin II to angiotensin) while having reduced plasma renin activity and aldosterone levels [17]. This finding was also observed by Spahic *et al*., where renin activity was significantly downregulated in POTS patients compared to healthy individuals. They also observed a significant inverse correlation between renin activity and supine and orthostatic blood pressure levels in healthy control group, but not in POTS patients. They concluded that renin activity in POTS patients is dissociated from supine and standing blood pressure levels in contrast to the control group [18].

The hemodynamic effects that would be expected with elevated AT II - high blood pressure and fluid retention - are absent in POTS patients. These data suggest that AT1R might be hyporesponsive to the effects of AT II in POTS patients [17]. One of the explanations could be the blocking effect or structural damage caused by AT1R antibodies. The AT1R antibodies mediated damage of ATR1 receptors could also explain the low levels of aldosterone in POTS patients.

Rabbit model in one study showed that injecting the AT1R antibodies into the organism disrupted vasoconstriction by decreased vascular response to AT II [7].

Mustafa *et al*. propose decreased adrenal responsiveness to chronically elevated levels of AT II. Similarly, chronic exposure to high levels of AT II could cause relative resistance to AT II because of the down - regulation of its receptors with reduced vasoconstriction on orthostatic challenge [16]. In another study performed by Mustafa *et al*. in 2012, they evaluated the responsiveness of target tissues, specifically the adrenal gland, to AT II infusions. They found that patients with POTS have blunted systemic vascular response to angiotensin II - they documented significantly smaller increment in mean arterial pressure in POTS patients compared with controls - while having a normal renal plasma flow, aldosterone secretion and sodium reabsorption by the kidneys. Another finding of their study was that POTS patients have impaired baroreflex sensitivity due to high circulating AT II compared to healthy controls. This could result in an excessive increase in heart rate in an upright position [17].

AT II can also act centrally to increase the sympathetic activity by binding to AT1R in the circumventricular structures of the brain (structures around third and fourth ventricle). AT II then facilitates neurotransmission through noradrenaline by both increasing its release and inhibiting its reuptake in the nerve terminals. Given the fact that POTS patients have increased levels of ATII (see above), this could be a possible mechanism explaining the tachycardia in POTS patients. Whether this mechanism contributes to high levels of noradrenaline in POTS patients is still unknown [16].

These findings suggest that abnormal regulation of the renin-angiotensin-aldosterone system may contribute to the pathophysiology of POTS [16]. Although simple AT1R finding could not discriminate POTS from not-POTS, the pathomechanisms of RAAS in POTS still need further studies. Also it is unknown whether the AT1R antibodies are pathogenic or simply elevated due to secondary mechanism,

Fedorowski et al performed research comparing the mean activity values of autoantibodies against ADRA1, ADRB1, ADRB2 between 3 groups - POTS group, VVS group and the control group. Among the 17 patients with POTS, 8 patients showed direct activation of ADRA1, 11 patients showed activation of ADRB1 and 12 of ADRB2 and none of the autoantibodies were present in VVS or control group. There was no significant difference between VVS and the control group [8].

Furthermore, Gunning et al, in their study with 55 POTS patients enrolled, found that most POTS patients have at least 1 elevated G-protein coupled adrenergic receptor antibody (alpha-1 and alpha-2 adrenergic receptors and beta-1 and beta-2 adrenergic receptors), and in some cases, both adrenergic and muscarinic antibodies. Although the main limitation of this study was the lack of the control group [13]. Dubey et al reported increased presence of muscarinic receptor type 1 and 2 antibodies in POTS patients compared to controls (study enrolled 16 POTS patients and 20 controls) [19].

We have not confirmed these results. In our study, autoantibody concentration against ADRA1, ADRB1, ADRB2 and M4R were not significantly different between POTS patients and controls or VVS patients.

On the other hand there are some studies that question the role of autoimmunity in POTS. Hall et al, in their study with 116 POTS patients and 81 controls, documented that autoantibody concentrations (AT1R, ETAR (endothelin type A receptor), ADRA1, ADRA2, ADRB1, ADRB2, and M1R through M5R) were not different in POTS patients and healthy controls. Majority of patients with POTS (90 %) and all healthy controls (100 %) were seropositive for ADRA1 [20].

Despite extensive research, there is no consensus as to what role these autoantibodies play in the pathophysiology of POTS [4, 6, 20]. The possible reasons for controversy and different results among studies evaluating levels of autoimmune antibodies in patients with POTS intolerance are different methods of antibody level measurement determination, different inclusion and exclusion criteria, possible confounding effect of concomitant medication and in general small number of patients.

The real role of the autoantibodies in the pathophysiology of POTS depends on their ability to structurally damage, block or activate a specific receptor, therefore functional assays are better suited to study the role of the autoantibodies.

The possible way how to study the role of autoantibodies is also to inactivate or remove specific antibodies in a therapeutic way. Answering the question if such an approach can improve the clinical manifestation of the disease could contribute to better understanding of the autoimmunity role in orthostatic intolerance syndromes [10, 21]. Considering the possible autoimmune aetiology of POTS, some studies investigated the effect of immunotherapy. To date, the published literature is limited to a small number of case reports documenting improvement in POTS symptoms after intravenous immunoglobulin treatment. Currently, immune-modulatory therapies in POTS are not recommended unless a systemic autoimmune disorder is confirmed [4,22].

## Limitations

In our study we compared only the levels of the autoantibodies between study groups. Functional assays would be able to evaluate the ability of autoantibodies to activate/block G-protein coupled receptors. Yu et al found that patients with POTS had significantly higher autoantibody activity to the angiotensin II type 1 receptor, even when the serum levels were similar in the POTS patient group and the control group [7].

## Conclusion

We conclude that the present study supports the autoimmune background of POTS. In our study, we found significantly higher levels of autoantibodies against angiotensin II type 1 receptor in POTS patients compared with healthy controls. Abnormal regulation of renin-angiotensin-aldosterone system may contribute to the pathophysiology of POTS. We have not found any significant difference in autoantibodies against ADRA1, ADRB1, ADRB2, or M4R receptors between POTS patients, VVS patients, and healthy control.

## Figures and Tables

**Table 1:** Demographic and clinical data

	POTS	VVS	Controls	P value
*Age (years)*	31.73±11.20	43.41± 17.19	41.81±15.58	0.12
*Females/males*	11/8	26/16	14/8	0.92
*Autoimmune disease*	2	2	0	0.29
*Diabetes mellitus*	0	1	0	0.61
*Arterial hypertension*	4	11	0	0.03
*Bronchial asthma*	1	2	0	0.56
*Thyroid gland disease*	4	5	0	0.09

POTS - postural orthostatic tachycardia syndrome; VVS - vasovagal syncope

**Table 2:** Levels of the M4R, ADRA1, ADRB1, ADRB2 and AT1R autoantibodies

	POTS	VVS	Control group	p-value
	n=19	n=42	n=22	
*M4R (U/ml)*	9.01±6.18	9.66±10.71	7.723±2.835	0.819
*ADRA1 (ng/ml)*	0.461±0.115	0.527±0.233	0.602±0.291	0.257
*ADRB1 (ng/ml)*	87.3±47.3	88.66±55.92	80.45±24.90	0.835
*ADRB2 (ng/ml)*	239.53±38.49	244.74±39.33	252.25±26.28	0.234
*AT1R (ng/ml)*	0.678±0.353	0.467±0.349	0.382±0.320	0.015

M4R - muscarinic receptor type 4; ADRA1 - alpha-1 adrenergic receptor; ADRB1 - beta-1 adrenergic receptor; ADRB2 - beta-2 adrenergic receptor; AT1R - angiotensin II type 1 receptor; POTS - postural orthostatic tachycardia syndrome; VVS - vasovagal syncope)

**Table 3:** Levels of the M4R, ADRA1, ADRB1, ADRB2 and AT1R autoantibodies in POTS patients and controls according to gender

	POTS men (n=7)	POTS women (n=12)	Control men (n=10)	Control women (n=12)	p-value
*M4R (U/ml)*	7.30±2.61	10.25±7.74	8.65±3.35	7.19±2.47	0.39
*ADRA1 (ng/ml)*	0.49±0.11	0.43±0.12	0.52±0.24	0.65±0.31	0.16
*ADRB1 (ng/ml)*	88.49±30.24	86.36±58.14	84.16±26.17	78.33±24.88	0.93
*ADRB2 (ng/ml)*	237.57±43.83	240.96±36.28	261.18±42.74	253.09±23.70	0.48
*AT1R (ng/ml)*	0.75±0.37	0.63±0.35	0.35±0.30	0.40±0.34	0.06

M4R - muscarinic receptor type 4; ADRA1 - alpha-1 adrenergic receptor; ADRB1 - beta-1 adrenergic receptor; ADRB2 - beta-2 adrenergic receptor; AT1R - angiotensin II type 1 receptor; POTS - postural orthostatic tachycardia syndrome)

**Table 4:** Levels of the M4R, ADRA1, ADRB1, ADRB2 and AT1R autoantibodies in VVS patients and controls according to gender

	VVS men (n=17)	VVS women (n=25)	Control men (n=10)	Control women (n=12)	p-value
*M4R*	7.08±1.74	9.88±11.61	8.65±3.35	7.19±2.47	0.62
*ADRA1*	0.51±0.19	0.58±0.27	0.52±0.24	0.65±0.31	0.56
*ADRB1*	76.34±22.03	86.37±63.01	84.16±26.17	78.33±24.88	0.89
*ADRB2*	253.48±53.24	238.96±27.65	261.18±42.74	253.09±23.70	0.46
*AT1R*	84.36±0.33	0.45±0.37	0.35±0.30	0.40±0.34	0.72

M4R - muscarinic receptor type 4; ADRA1 - alpha-1 adrenergic receptor; ADRB1 - beta-1 adrenergic receptor; ADRB2 - beta-2 adrenergic receptor; AT1R - angiotensin II type 1 receptor; VVS - vasovagal syncope)
